# Supplementary figures and images for: Multi-Omics Alleviates the Limitations of Panel Sequencing for Cancer Drug Response Prediction
Source: Cancers (Basel). 2022 Nov 15;14(22):5604. doi: 10.3390/cancers14225604 (PMC9688044; doi:10.3390/cancers14225604)

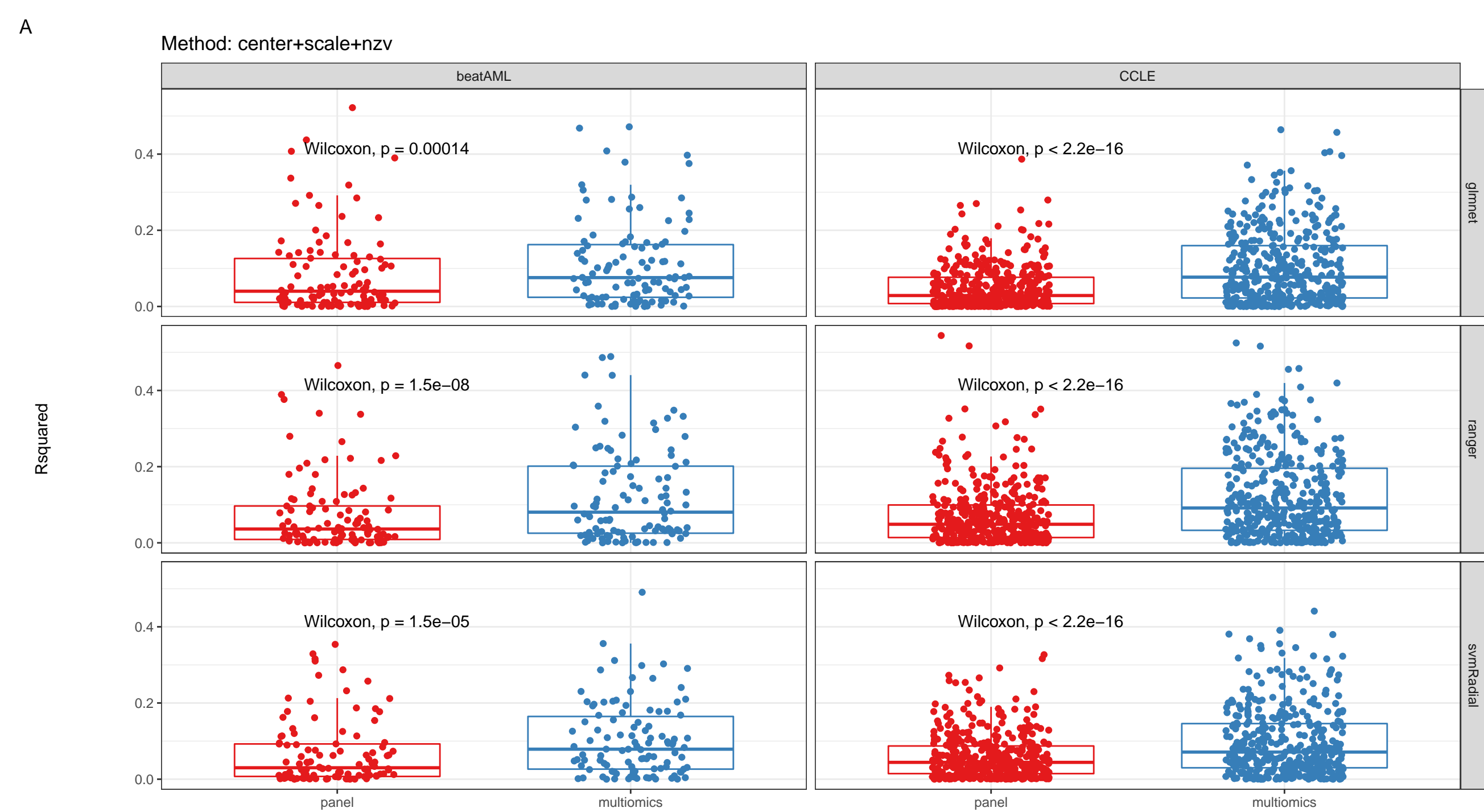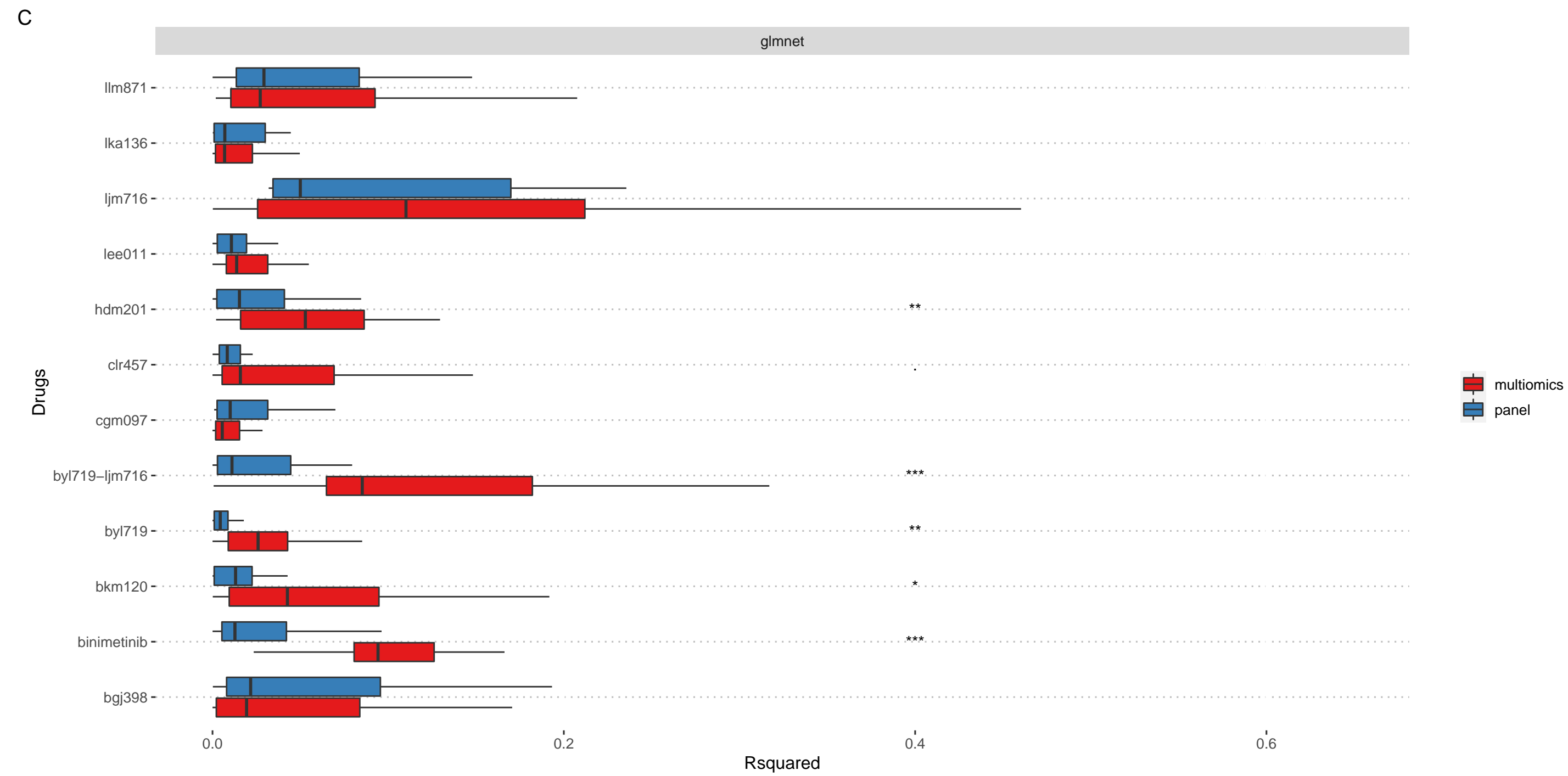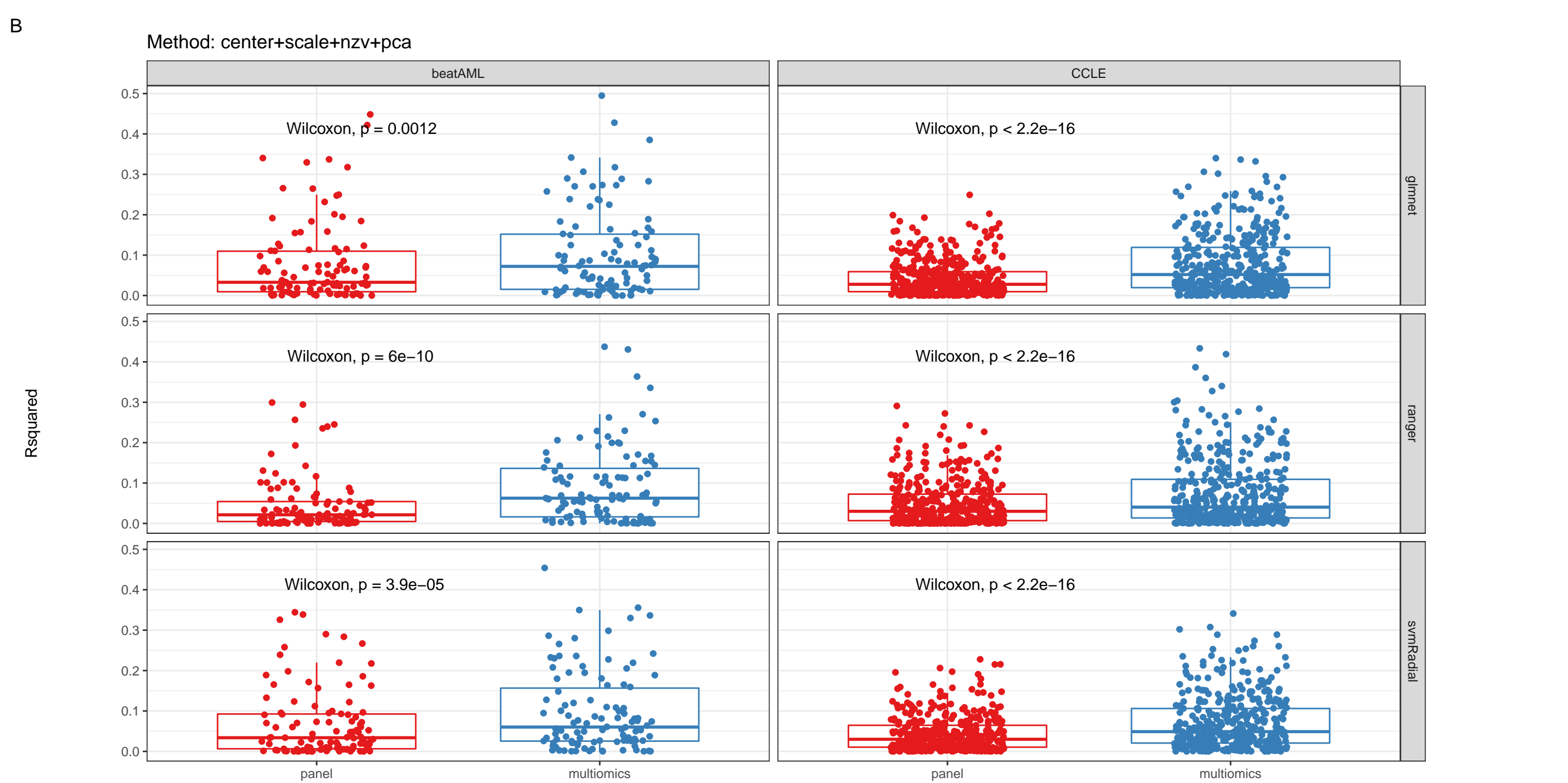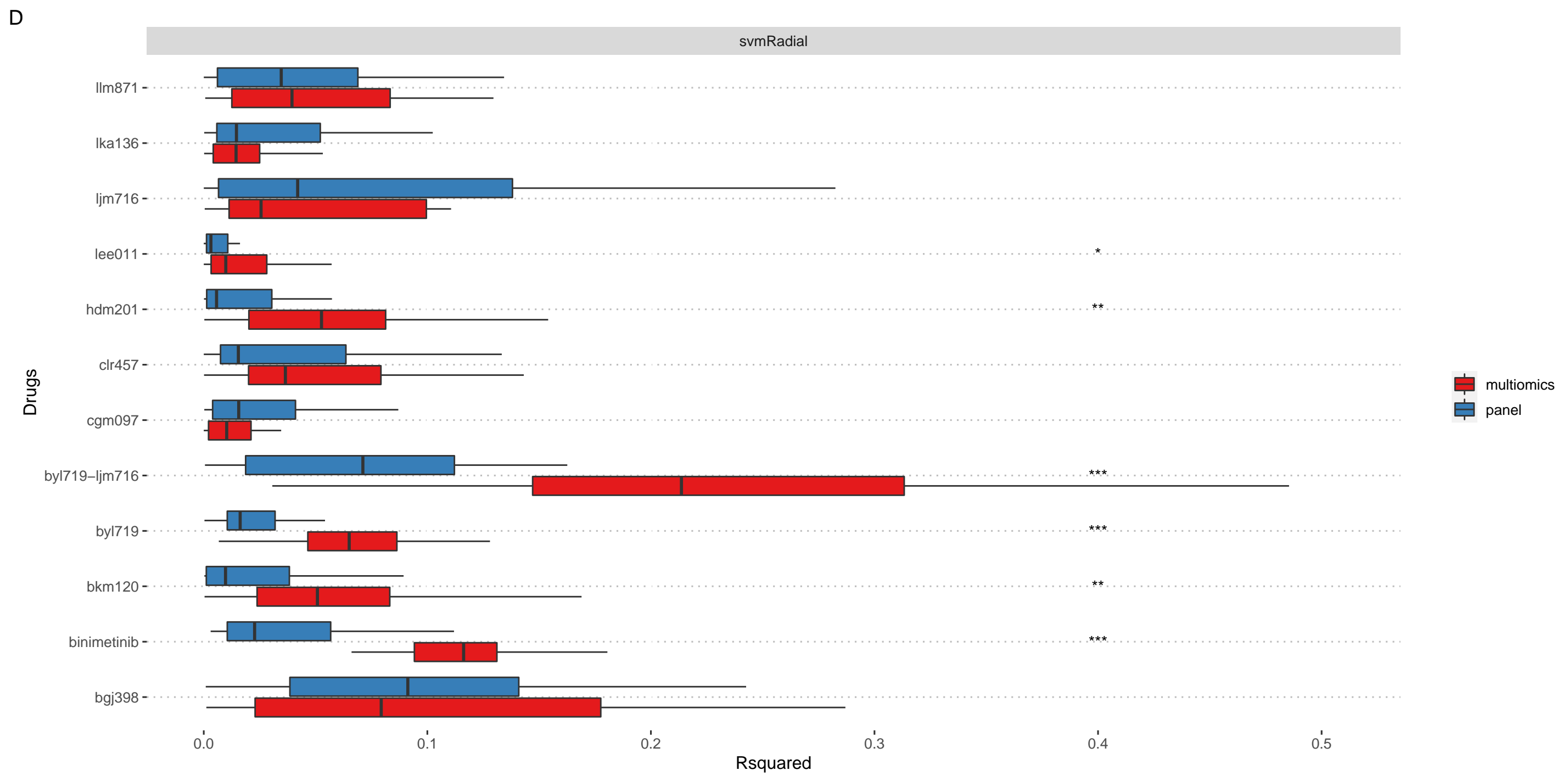

Supplement: Supplementary file 1 [file cancers-14-05604-s001.zip › figure S1.pdf]

**A**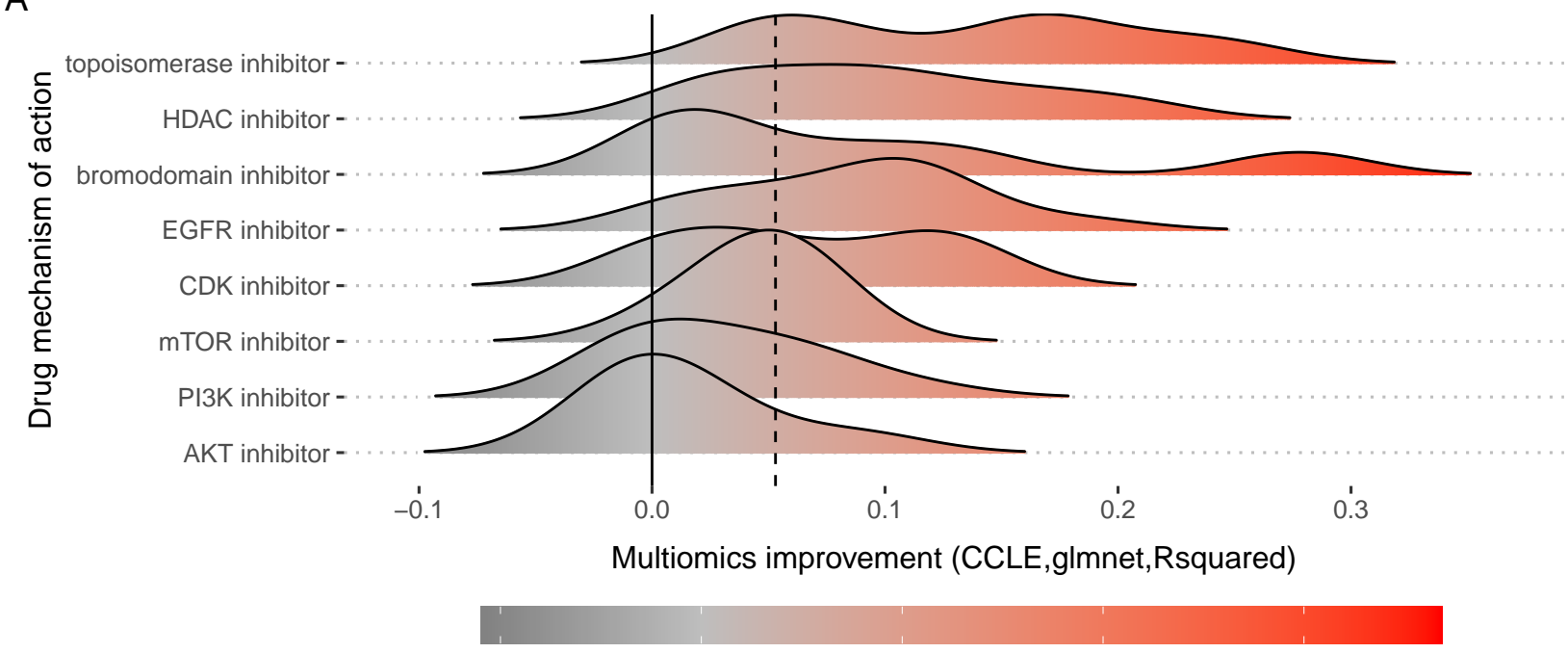**B**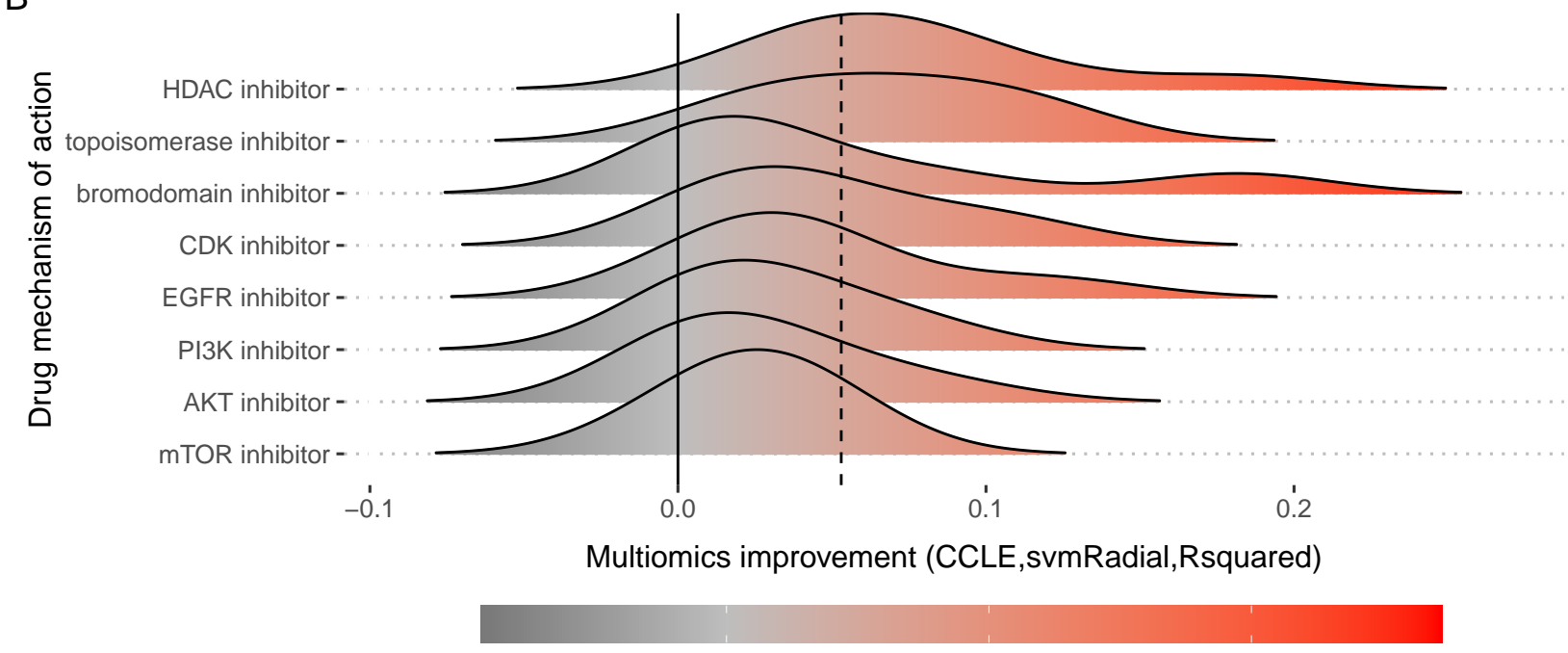**C**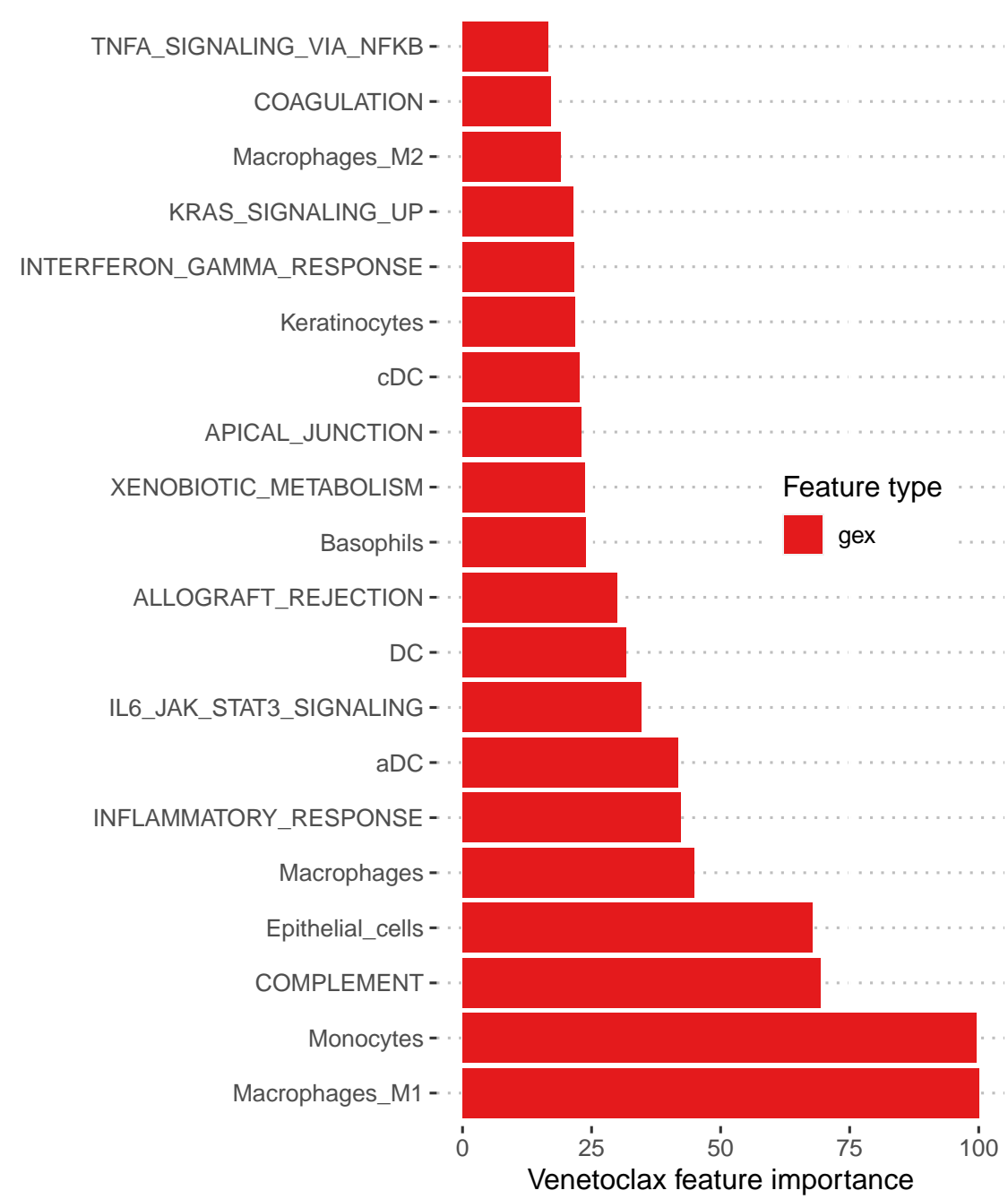

Supplement: Supplementary file 1 [file cancers-14-05604-s001.zip › figure S2.pdf]
